# Recombination Pattern Characterization via Simulation Using Different Maize Populations

**DOI:** 10.3390/ijms21062222

**Published:** 2020-03-23

**Authors:** Wei Ren, Xiaoping Gong, Kun Li, Hongwei Zhang, Fanjun Chen, Qingchun Pan

**Affiliations:** 1 College of Resources and Environmental Sciences; National Academy of Agriculture Green Development; Key Laboratory of Plant-Soil Interactions, Ministry of Education, China Agricultural University, Beijing 100193, China; renwei2012@126.com (W.R.); littlepgong@163.com (X.G.); caucfj@cau.edu.cn (F.C.); 2 Institute of Crop Sciences, Chinese Academy of Agricultural Sciences, Beijing 100081, China; likun01@caas.cn (K.L.); zhanghongwei@caas.cn (H.Z.)

**Keywords:** maize, simulation, recombination, doubled haploid, population

## Abstract

Efficient recombination is critical to both plant breeding and gene cloning. However, almost all traditional recombination studies and genetic improvements require the slow and labor-intensive population construction process, and little is known about the recombination characteristics of populations of different types, generations, and origins. Here, we provide a simple and efficient simulation method for population construction based on doubled haploid (DH) and intermated B73 × Mo17 maize (IBM) populations to predict the recombination pattern. We found that the chromosomes had 0, 1, 2, and 3 recombination events that occurred at rates of 0.16, 0.30, 0.23, and 0.15, respectively, in the DH and the recombination rate of each chromosome in the IBM population ranged from 0 to 12.1 cM per 125 kb. Based on the observed recombination parameters, we estimated the number of recombination events and constructed the linkage maps of the simulated DH and recombination inbred line (RIL) populations. These simulated populations exhibited similar recombination patterns compared with the real populations, suggesting the feasibility of this simulation approach. We then compared the recombination rates of the simulated populations of different types (DH induced or self-crossed), generations, and origins (using the 8, 16, and 32 multiparent advanced generation intercross (MAGIC) populations), and suggested a rapid and cost-effective population construction procedure for breeders and geneticists, while maintaining an optimal recombination rate. This study offers a convenient method for optimizing the population construction process and has broader implications for other crop species, thereby facilitating future population studies and genetic improvement strategies.

## 1. Introduction

Recombination is the main driving force of genome evolution and genetic diversity [1]. During this process, double-strand breaks (DSB) are generated during meiosis and crossover events are formed upon DNA repair. The crossover frequency in different regions is controlled by stable genetic factors including both genic and intergenic elements [2,3]. To facilitate crop breeding and gene cloning, several aspects of recombination need to be more well-understood, including the recombination patterns of different generations and populations [4]. Recombination pattern is known to vary significantly among different populations, including the doubled haploid (DH) population, recombination inbred lines (RILs), intermated B73 × Mo17 maize (IBM) population, and multiparent advanced generation intercross (MAGIC) population. Although recombination studies have been previously performed in these populations, the influences of population types and generations on recombination patterns are largely unknown [5,6].

One of the most common methods to identify causal genes involves crossing two lines and screening recombinants for the phenotype of interest. The speed of this process largely depends on the recombination frequency within the area of interest, yet information about the relative frequency across the genome is mostly unknown [7,8]. Thus, a better understanding of recombination pattern will speed up the breeding process and improve efficiency. This could be accomplished through a targeted program of crossing over and maintaining elite genotypes by using recombination information of different generation and populations [4].

Doubled haploids (DHs) are generated through a process of inducing heterozygote materials to haploids and then doubling them into homozygous plants. Several major genes controlling maize DH induction have been cloned and utilized in wheat to improve DH induction efficiency [9,10]. The DH technology is now well-established and has greatly improved population construction efficiency [11]. The DH approach quickly converts heterozygous materials to completely homozygous lines and can be applied to various generations and populations [12].

The intermated IBM population has been employed in genetic mapping and quantitative genetic studies. The IBM population is derived by intercrossing F_2_ and F_3_ family lines and then self-crossing (CS) them to pure inbred lines [13]. Although there are a significant number of recombination events, generating this population is very labor- and time-intensive. Recently, an IBM-DH population was developed and used in a deep-sowing germination quantitative trait loci (QTL) study [14]. Compared with populations generated by DH induction in early generations, the IBM-DH population is relatively fast and cost-effective to generate, while enhancing QTL mapping resolution. The IBM population provides us with valuable information about recombination parameters, which can be used to help predict recombination characteristics in other populations.

With the development of population genetics and statistical methods, linkage analysis considering only two alleles can no longer meet the needs of genetic research. Integrated populations involving multiple parents are better suited to identify important variations. MAGIC populations have been utilized in recent years and are considered as a potential tool for future genetic studies and breeding [12]. The most important advantage of MAGIC populations is the increased number of parental alleles and recombination events, which can enhance mapping efficiency. In addition, MAGIC populations can also help breeders to select inbred lines with more favorable alleles [15]. MAGIC construction by the DH method is one possible solution to reducing the time and efforts required for population construction and the selection of desired progeny.

With the development of the DH technology, the recombination information of each line within the population could be accurately obtained [16]. Then, recombination patterns could be estimated by assessing the number of recombination events and overall recombination rate of the whole population [17], which allows geneticists and breeders to better determine population generations and types for improved experiment design and optimized population construction strategies. In this study, we simulated the recombination process of maize populations of different types and generations using two parameters—the number of recombination events and recombination rate. We also estimated the recombination pattern in populations of different types and generations, and in DH populations induced at different times, with implications for future genetic research and breeding.

## 2. Results

### 2.1. Variance of Two Key Recombination Parameters

**Figure 1:**
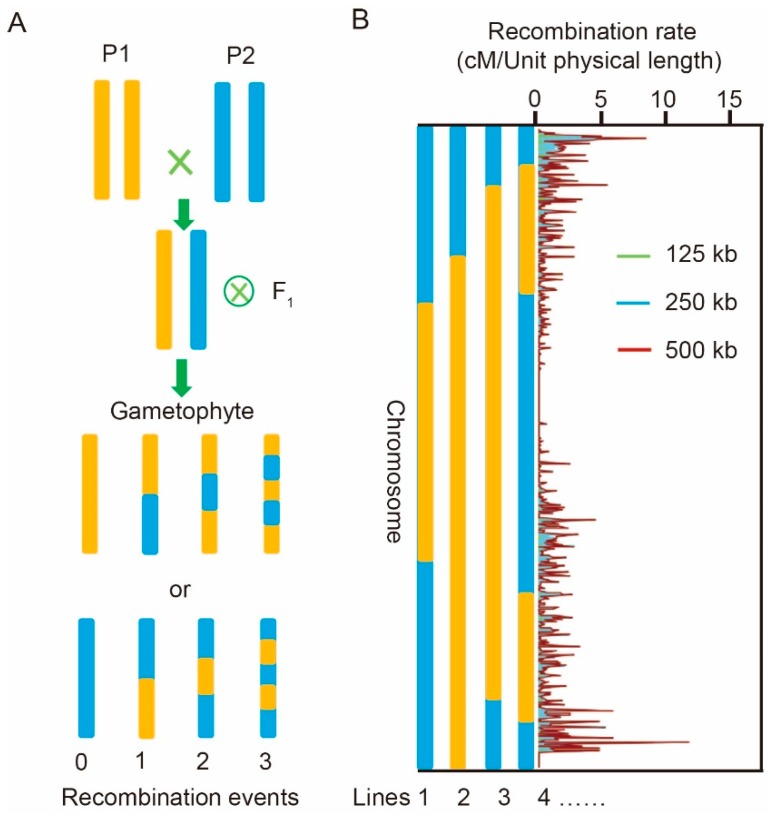
The recombination estimation process. (**A**) Estimation of the number of recombinant events of each chromosome in each line. (**B**) Estimated recombination rate of each chromosome based on the genetic map of the intermated B73 × Mo17 maize (IBM) population.

We first used the real genotypic data of the F_1_-doubled haploid (DH) and IBM populations to extract two recombination parameters. Recombination pattern is mainly determined by two parameters—the number of recombination events per chromosome and recombination rate of each site on the chromosome (Figure 1A). In the F_1_ generation, the number of recombination events can be determined in the F_1_-DH population or by comparing the parents’ genomes. In the present study, we evaluated the number of recombination events of each chromosome in each line using 23 maize DH populations containing 2233 F_1_-DH inbred lines (Supplementary Figure S1). The chromosomes contained 0, 1, 2, and 3 recombination events that occurred at rates of 0.16, 0.30, 0.23, and 0.15, respectively. The recombination rate of the IBM population (Figure 1B) varied considerably between 0 and 12.1 cM with an average of 3.48 cM, 3.74 cM, and 4.35 cM for 125 kb, 250 kb, and 500 kb windows, respectively. There were variations among the recombination rates of the three windows, but the recombination rate accuracy was higher in the 125 kb window (Supplementary Table S1). We combined the recombination event and rate parameters via an R-script, which enabled us to run simulations of various population types.

### 2.2. Comparison between Simulated and Real Genotypic Data

**Figure 2:**
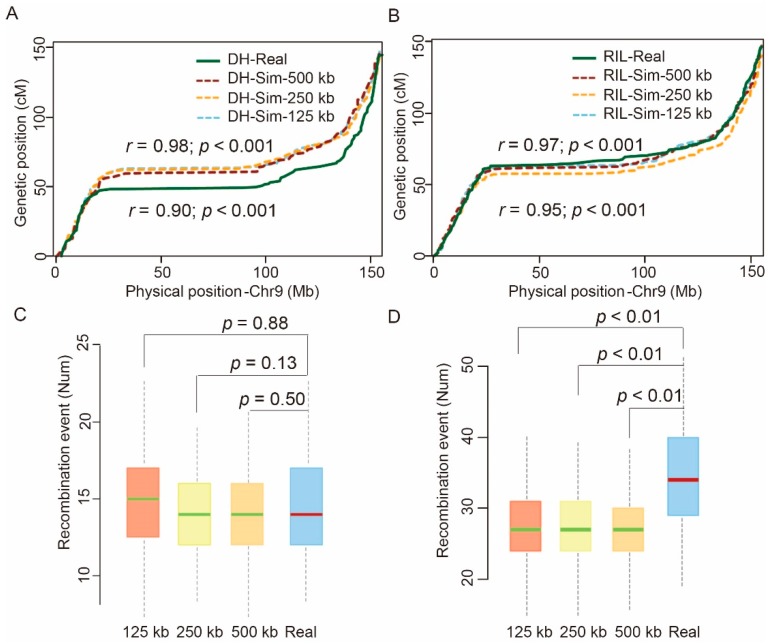
Comparison of the recombination pattern between the simulated and real doubled haploid (DH) and recombination inbred line (RIL) populations. (**A**) Comparison of the chromosome 9 linkage maps drawn based on the simulated and real DH populations under different window sizes. (**B**) Comparison of the chromosome 9 linkage maps constructed based on estimated and real RIL populations under different window sizes. Correlation analysis was performed to evaluate the consistency based on the recombination rate value within 125 kb interval between the simulated physical and genetic maps and those generated based on the real data. Comparisons were made between the simulated genetic maps of 125 kb and 500 kb, and between the simulated genetic map of 125 kb and the real genetic map. (**C**) Recombination patterns of the simulated and real DH populations under different window sizes (*n* = 200). (**D**) Consistency analysis of the average number of recombination events of each line between the simulated and real RIL populations under different window sizes (*n* = 200). Analysis of variance (ANOVA) was used to assess whether there was significant correlation between the simulated and real data.

To evaluate the accuracy of this simulation strategy, we then simulated the genotypes of 200 lines from each of the F_1_-DH and RIL populations using the recombination information described above. We also obtained the real genotypic data of 200 lines in each of the previously reported F_1_-DH and RIL maize populations [16,18]. We then compared the recombination information between the corresponding simulated and real populations to verify the accuracy of our simulation method. The linkage maps constructed based on F_1_-DH simulation of 125 kb and 500 kb exhibited consistent physical and genetic uniformity under all window sizes (*r* = 0.98; *p* < 0.001). The variance across different chromosome locations was also consistent between the two linkage maps of simulation (125 kb) and real data (*r* = 0.90; *p* < 0.001) (Figure 2A). An analysis with the 200 RILs yielded similar results—consistent physical and genetic orders were obtained by the simulated and real data under different window sizes (*r* = 0.95; *p* < 0.001; Figure 2B). The average number of recombination events of both the simulated and real DH population was 14 ± 3 (Figure 2C). By contrast, in the RIL population, we observed a discrepancy between the average number of recombination events based on the simulated (26 ± 6) and actual data (34 ± 7) (Figure 2D). The relatively higher number of recombination events in the RIL population was likely a result of the enrichment of small recombination fragments by eight rounds of continuous self-crossing. Taken together, these results indicated that the selected parameters were reliable, and the simulation method performed well in evaluating recombination efficiency in populations of different generations and types.

### 2.3. Recombination Pattern in DH Populations Induced at Different Generations

**Figure 3:**
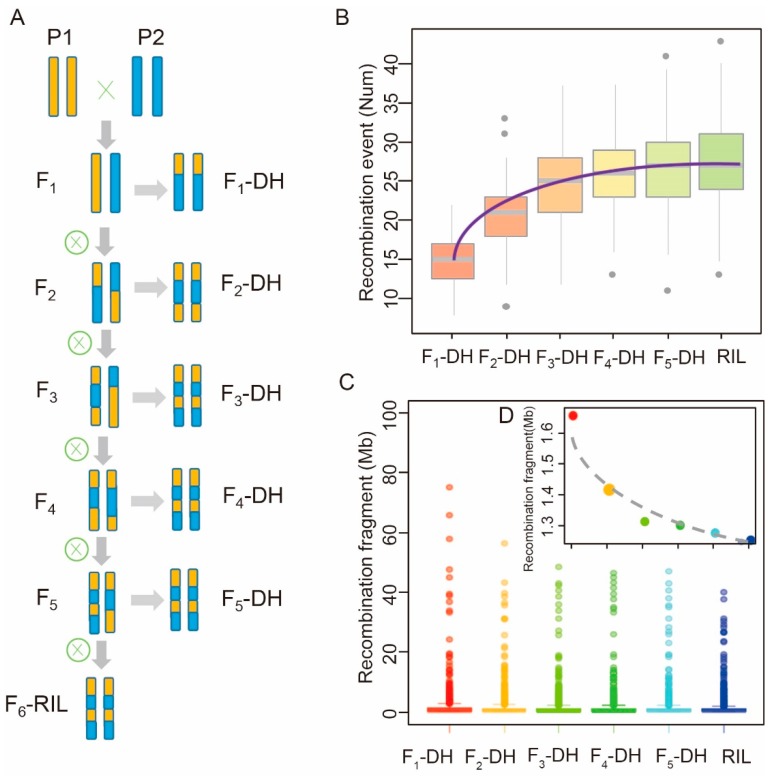
Recombination estimation of the RIL and DH populations. (**A**) Schematic of simulated construction processes of the RIL and DH populations of different generations. (**B**) A boxplot of the predicted recombination events of the abovementioned populations. The results of 125 kb window are shown, *n* = 200. (**C**) Predicted recombination fragment size of the RIL and DH populations of different generations. The results of 125 kb window are shown, *n* = 200.

We then assessed the influence of induction time (generation) of DH populations on the recombination pattern (Figure 3A). The results showed that, in general, the average number of recombination events increased from 14 to 27 from F_1_ to F_n_. The increase of the proportion of recombination events became slower with increasing generations and was negligible after F_6_. DH induction was the most efficient in the F_3_ generation, with a total recombination rate of up to 92.6% in the RIL population (Supplementary Table S2). The number of recombination events in F_3_-DH reached 24, followed by a slow increase afterward (Figure 3B, Supplementary Table S2). The length of recombination fragments ranged from 0.25 Mb to 75.0 Mb, with an average length of less than 1.66 Mb from F_1_ to F_6_. Additionally, centromeric regions tended to have larger recombination fragments than other regions (Figure 3C, Supplementary Table S2). The standard deviation of recombination fragment ranged from 4.6 Mb to 3.0 Mb from F_1_ to F_6_. Moreover, the median of recombination fragment length decreased from 0.62 Mb in F_1_-DH to 0.49 Mb in F_6_-DH (Figure 3D, Supplementary Table S2). The total number of recombination bins—blocks with no recombination—of the DHs induced at different generations varied from 1367 to 1858, with F_3_-DH showing the highest number of recombination bins. Collectively, these data show the feasibility of our method in estimating recombination during DH population construction and suggest that the F_3_ generation is the most optimal time for DH induction.

### 2.4. Recombination Pattern Comparison of MAGIC Populations Constructed by DH Induction and Continuous Self-Crossing

**Figure 4:**
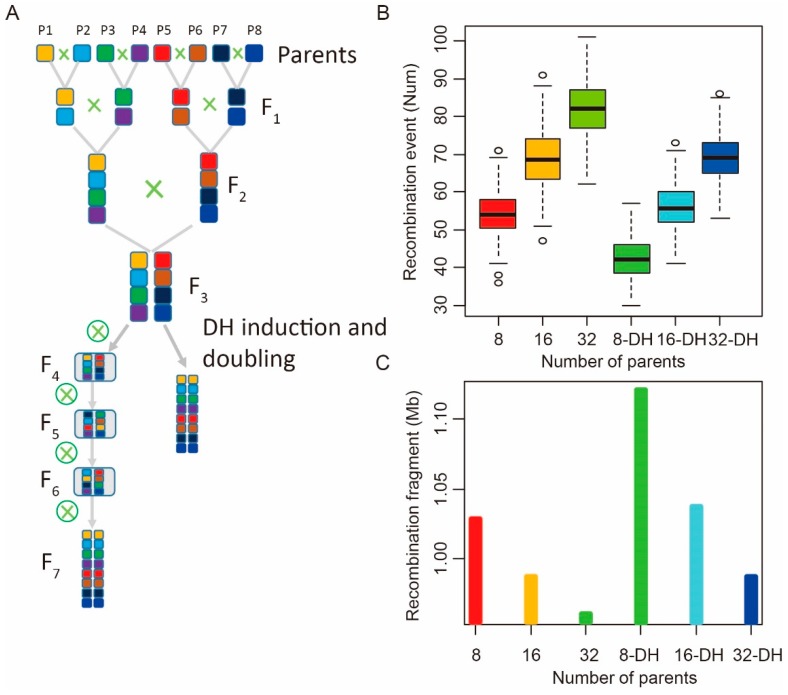
Recombination estimation of the 8, 16, and 32 multiparent advanced generation intercross (MAGIC) populations generated by DH induction and continuous self-crossing. (**A**) Schematic of the eight-parent MAGIC population construction. (**B**) Estimated recombination event and (**C**) average recombination fragment length of the 8-, 16- and 32-parent MAGIC populations derived by DH induction and continuous self-crossing, *n* = 200.

In addition to the biparental DH-induced populations, the maize MAGIC population has gained momentum in recent years because it contains multiple parental alleles and has a better mapping resolution. Here, we simulated and compared the recombination characteristics of the MAGIC population derived from 8, 16, and 32 parents constructed by two different methods—the DH technique and continuous self-crossing (Figure 4A, Supplementary Figure S2). According to our results, the average number of recombinant events of the DH-MAGIC populations (42–69) was lower than that of the CS-MAGIC populations (55–82). Average fragment lengths were similar between the DH-MAGIC (1.12–0.99 Mb) and CS-MAGIC (1.03–0.96 Mb) populations. However, it took eight to ten generations to construct the CS-MAGIC population, but only four to six generations to build the DH-MAGIC population. These finding suggest that, despite a slightly lower recombination rate, the DH-MAGIC populations are faster to build, thereby having a higher efficiency. Subsequent analyses further supported this notion. For example, the number of single crossover events increased with increasing number of parental lines in populations built by both the DH and CS methods. Specifically, 2314, 2431, and 2516 recombination bins were estimated in the 8-, 16-, and 32-parent CS-MAGIC populations, respectively; these numbers were higher than that predicted for DH-induced MAGIC populations, which were 2122, 2289 and 2433, respectively (Supplementary Table S3). Fragment length was similar between populations constructed by the two methods (Figure 4C, Supplementary Table S3). Considering that the recombination rate of the 16-parent CS population was comparable to that of the 32-parent DH population, we conclude that the DH method is more efficient because it requires less time to generate (Figure 4B).

## 3. Discussion

### 3.1. Feasibility and Influencing Factors of the Simulation Method

In the past, researchers had to construct populations to study recombination, which was difficult to do in a high-throughput manner. Recombination characteristics of the populations can now be predicted computationally. Stumpf and McVean (2003) have proposed that the number of recombination events and recombination rate are the most crucial parameters for predicting recombination patterns [17]. Therefore, we used these two parameters to simulate recombination patterns in populations of different types (DH and CS), generations, and origins (biparental vs. multiparental populations). The feasibility of this method was verified by comparing the results with the real data. The simulation method proposed here is fast, cost-effective, and applicable to different generations and population types.

In the current study, we only considered the number of recombination events and recombination rate, although other factors may also play minor roles. Recombination events and recombination rate are regulated by the mechanism in vivo. Other factors mainly influence recombination by indirectly influencing the internal mechanism of recombination. Recombination events are mainly determined by genes, which has been confirmed in previous studies [19]. The variation of the recombination rate was mainly affected by local genomic elements. These two factors are controlled by the internal mechanism, and they are very stable genetic mechanisms [3]. Other factors have little effects on recombination, mainly because they modify the mechanism of recombination, which has been confirmed in previous research [1]. This may explain why the recombination rate of the simulated populations was slightly lower than that calculated by the real data [3]. Additionally, we only took into consideration the number of crossover (CO) events in this study, whereas small recombination fragments generated by other mechanisms, such as gene conversion and double crossing over, may have resulted in the underestimation of recombination rates [20,21]. In real case scenarios, the number of recombination events is also affected by environmental factors that are not considered by the simulation method [22,23]. Moreover, a recent publication has reported that paternal genotypes can also affect the number of recombination events [24]. Besides the aforementioned factors, chromosome length is another important influencing factor of recombination frequency [25]. Thus, future simulation studies on recombination characteristics should incorporate these variables to achieve a higher predictive power.

### 3.2. Recombination Simulation Is a Fast and Cost-Effective Approach for Genetic Studies and Crop Improvement

Previous studies on recombination often involve the long and labor-intensive population construction process and the sequencing of all lines and individuals within them. Here, we offer a novel and cost-effective DH-based simulation strategy for studying recombination characteristics. To the authors’ knowledge, this is the first report of estimating population size and recombination patterns by simulation. 

The DH method has emerged as a useful tool that has been widely employed by researchers and breeders to accelerate the population construction process. However, some researchers have expressed their concerns about the low recombination efficiency of DH populations [12]. Therefore, we assessed the feasibility of our method by comparing the recombination efficiency of populations generated by the DH and canonical CS strategy. We first determined that the F_3_ generation is the best time for DH induction after assessing the recombination efficiency in populations induced in the F_1_ to F_5_ generations. Then, we investigated how the number of parents affected recombination frequency. The results showed that the estimated recombination rate of the 32-parent MAGIC population constructed by the DH method was comparable to that of the 16-parent MAGIC population generated by the CS strategy. Considering that the DH method is 2–3 generations faster than the traditional CS method to achieve similar recombination rates, we believe that DH induction can be utilized as a rapid and reliable strategy to accelerate population construction. 

Breeding pyramids favor alleles from elite germplasms and select progenies with the most optimal phenotypic expression to take forward. Active recombination is a prerequisite for breeding, and an enhanced recombination frequency will facilitate linkage drag (a major hinderance of recombination) breakage and favorable allele accumulation. The DH strategy has been successfully employed in the genetic study and breeding of maize and other major crop species [26]. Despite the importance and wide application of the DH strategy, there is a dearth of scientific studies investigating recombination characteristics of the DH populations. Here, we simulated the recombination pattern in populations of different generations and types using two major recombination parameters, aiming to provide guidance for optimizing the population construction process. Specifically, we observed an exponential increase in the number of recombination events and a decrease in the length of recombination fragments in DH populations induced in the F_3_ generation compared to that induced in the F_1_ generation. By contrast, from the F_4_ generation onward, the improvements in these two parameters were marginal. We propose the following steps for commercial breeding programs because early determination (F_2_–F_4_) of general combining ability (GCA) is crucial for conventional breeding. First, GCA determination should be performed in the F_2_ generation of biparental populations and progenies with low GCA should be discarded. Next, DH induction of high GCA families should be carried out in the F_3_ generation. This procedure can greatly facilitate crop breeding due to its high efficiency and low cost.

Employing multiple parents in population construction for recurrent selection and genetic improvement has the benefit of enhancing the genetic gain and even achieving a qualitative leap in some traits [12]. The MAGIC population is a simulated self-crossing population, whose construction can be extremely labor-intensive and time-consuming. This study offers a rapid and cost-effective way of constructing a 32-parent MAGIC population using the DH method, while maintaining a recombination rate comparable to that of the 16-parent MAGIC population generated by the traditional CS method. The simulation method reported in this study will greatly facilitate population genetic studies and crop improvement.

### 3.3. The Simulation Method Has Broader Implications

The simulation method presented here has wide application prospects in other crop species, such as rice and *Triticum*. The rice genome sequences are of better quality compared with that of maize, which allows for a better estimation of the recombination parameters as well as easier population construction and evaluation [27,28]. In addition, the two simulation parameters we selected are at the chromosomal level, and are thus less affected by the complexity of the genome, enabling the application of this simulation method in polyploids, such as the hexaploid wheat [29].

Gene mining and cloning provide valuable genetic resources for breeding and the genetic improvement of crop species. With the advancement of QTL mining strategies, such as the bulk segregant analysis (BSA) of MutMap, QTL-seq, MutMap-Gap, bulked segregant RNA-seq (BSR-seq), and new quantitative trait gene sequencing (QTG-seq), estimating the appropriate population size required for the desired mapping resolution has become a new challenge [8,30,31,32,33,34]. The recombination estimation method presented here offers guidance for population construction design and population size determination. It is also applicable to a wide variety of species and breeding targets, and will benefit genetic research and crop improvement in the long run.

## 4. Materials and Methods

### 4.1. Determination of the Two Recombination Parameters

We determined the number of recombination events following previously reported methods used for F_1_-DH populations [16,35]. First, the number of recombination events of each chromosome was calculated in each F_1_-DH line of the 23 DH populations (containing 2233 lines in total). The number of recombination events is defined as the number of break points in each chromosome in each line. The recombination rate of each population was calculated by dividing the total number of recombination events of the whole population by the number of lines. This allowed us to estimate the number of recombination events of each generation on the chromosome level. The recombination pattern of the maize IBM populations was calculated by dividing the number of recombination events within 125 kb, 250 kb, and 500 kb intervals by the number of lines within each population. The recombination probability was calculated by dividing the recombination rate of each window size by the overall recombination rate of each chromosome. These two parameters were used to analyze recombination patterns in populations of different types and DH induction times. A random walk for gametes transmission from different parents was considered. Crossovers by neighboring loci and linkage phase of double recombination on the same chromosome with physical distance larger than 10 Mb were selected. The R-script of this simulation method is available at https://github.com/panqingchun/recombination_simulation/.

### 4.2. Estimation of the Number of Recombination Events and Fragment Length

The DH (F_1_-DH to F_5_-DH) and RIL populations were simulated in this study, using the two parameters mentioned above. Two hundred lines from each generation of the simulated population were randomly produced. We first designated the two parents as P1 and P2, and crossed them to generate F_1_. The recombination pattern of the F_1_-DH population was predicted based on the two parameters using the R-script. The recombination probability under 125 kb, 250 kb, and 500 kb windows was computed. The same procedure was repeated for the F_2_-DH to F_5_-DH populations. Similarly, the RIL population was simulated by six generations of self-crossing via the single seed descent method. Each chromosome’s information was translated to the next generation. The number of recombination events of all ten chromosomes was computed by predicting recombination breakpoints. The size of the recombination fragment was calculated as the distance between two neighboring breakpoints. The number of recombination events and fragment size were calculated by the following formula:

(1) $$\mathrm{Number}\;\mathrm{of}\;\mathrm{recombination}\;\mathrm{events}/\mathrm{line}=\overset{10}{\underset{k=1}{\sum }}\mathrm{Number}\;\mathrm{of}\;\mathrm{recombination}\;\mathrm{break}\;\mathrm{point}\;\mathrm{on}\;\mathrm{chromosome}\;k$$

(2) $$\mathrm{Recombination}\;\mathrm{fragment}\;\mathrm{length}/\mathrm{line}=\frac{\mathrm{Physical}\;\mathrm{length}\;\mathrm{of}\;\mathrm{all}\;\mathrm{chomosomes}}{\mathrm{Number}\;\mathrm{of}\;\mathrm{recombination}\;\mathrm{break}\;\mathrm{points}}$$

ANOVA was used to analyze the differences in the number of recombination events and fragment length among populations. The simulated data were compared with the real genotype data of two previously reported populations—the real F_1_-DH and RIL populations [16,18]—to validate the feasibility of the simulation method.

### 4.3. Comparison of the Linkage Maps of the Simulated and Real Populations

The linkage maps, drawn based on the real and simulated data, were compared in a similar manner. We employed the “IciMapping” software to estimate the length of the linkage map based on the physical positions of the B73 reference genome [36]. The length of F_1_-DH and RIL linkage maps was predicted using the map function “kosambi” implemented in “IciMapping” by setting population types as “DH” and “RIL”, respectively. Linkage maps of the aforementioned real F_1_-DH and RIL populations were constructed based on data obtained from public databases [16,18]. We set the heterozygous genotype to zero in an in-house Perl script and filled the missing genotype data points with that of the most adjacent genetic marker. A correlation analysis was used to analyze the physical and genetic consistency of the simulated and real genetic maps.

### 4.4. Analysis of the Recombination Pattern in Different MAGIC Populations

As shown in Figure 4 and Supplementary Figure S2, we simulated the construction of 8-, 16-, and 32-parent MAGIC populations via DH induction and continuous self-crossing. To simulate the construction of the 8-parent MAGIC population by DH induction, we first crossed the eight parents to generate four F_1_s and then crossed the resulting F1s to generate two F2s, followed by crossing the two F_2_s to generate one F_3_. The resulting F_3_ was DH-induced to obtain F_3_-DH lines, which were then continuously self-crossed for five generations to generate an F_8_-DH-MAGIC population. The 16- and 32-parent DH-MAGIC populations were generated in a similar manner, and DH induction was carried out in the F_4_ and F_5_ generations to generate the F_9_-DH-16-parent MAGIC and F_10_-DH-32-parent MAGIC populations, respectively. Two hundred lines from each of the resulting populations derived by DH induction and CS were analyzed for the number of recombination events and recombination fragment length by simulation. The number of recombination events and fragment size with different parents and types of MAGIC populations were calculated by the abovementioned Equations (1) and (2).

### 4.5. Code Available

Codes for estimating the F_1_- to F_5_-DHs, RILs, as well as the 8-, 16-, and 32-parent MAGIC populations generated by DH induction and CS are available at http://github.com/panqingchun/recombination_simulation/.

## 5. Conclusions

In this study, recombination event and rate were used to estimate recombination patterns of different population types and generations through simulation analysis. Our result showed that biparental DH population induced in F3 generation had optimal recombination utility efficiency. Furthermore, by comparing the recombination utility efficiency of MAGIC populations constructed using different methods (DH induction or continual self pollination), we found that DH induction method had a higher efficiency and required shorter time to construct MAGIC populations. Our study proposed a fast and cost-effective method for optimizing the population construction process.

## Supplementary Materials

Supplementary materials can be found at https://www.mdpi.com/1422-0067/21/6/2222/s1.

## Abbreviations

BSA	Bulk segregant analysis
BSR-seq	Bulked segregant RNA-seq
CO	The number of crossover events
CS	Self-crossing
DSB	Double-strand breaks
DH	Doubled haploid
GCA	General combining ability
IBM	B73 × Mo17 maize population
MAGIC	Multiparent advanced generation intercross populations
QTG-seq	Quantitative trait gene sequencing
QTL	Quantitative trait locus
RIL	Recombination inbred line
