# Supplementary material for: Recombination Pattern Characterization via Simulation Using Different Maize Populations
Source: Int J Mol Sci. 2020 Mar 23;21(6):2222. doi: 10.3390/ijms21062222 (PMC7139635; doi:10.3390/ijms21062222)
Supplement: Supplementary file 1 [file ijms-21-02222-s001.pdf]

Supplementary Materials

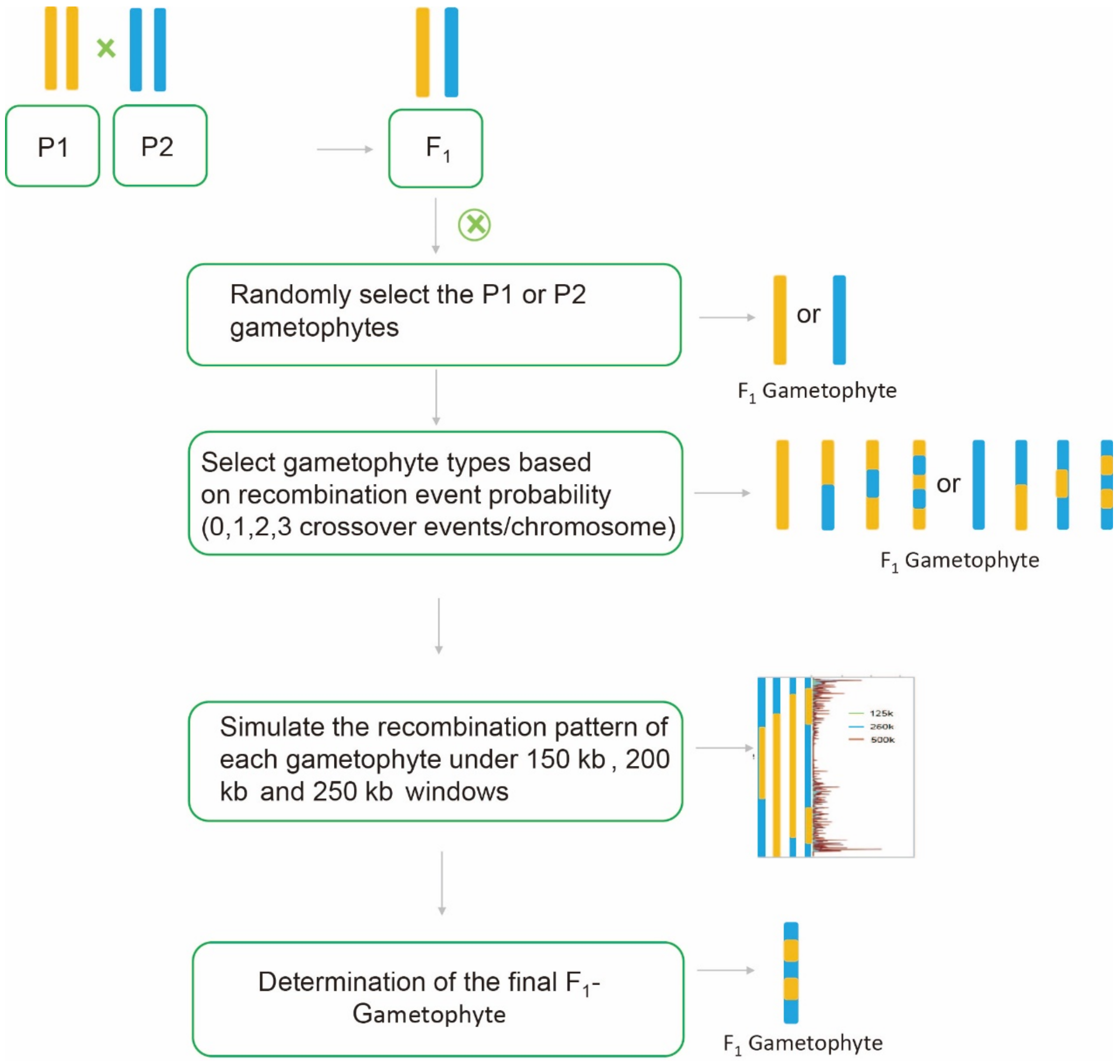

Figure S1. Overview of the recombination pattern estimation process. Assessment of the recombination process using the parental F_1-DH map and IBM genetic linkage map. First, two pure parental lines were crossed to generate an F_1, and P1 or P2 gametophytes were randomly selected based on recombination probability to estimate the number of recombination events per chromosome in the F_1-DH population. We then predicted the recombination rate of each location based on the IBM population. Finally, the recombination pattern of each subsequent generation was simulated by an integrated analysis of the two recombination parameters.

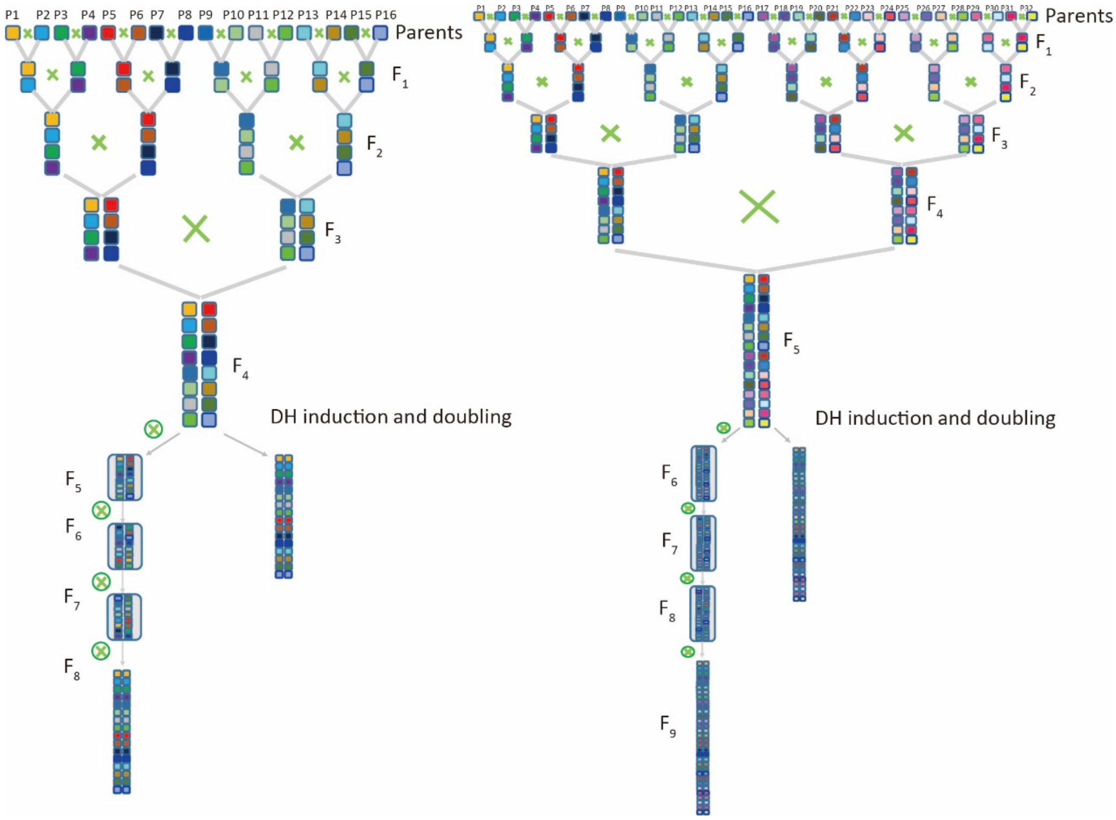

Figure S2. Construction of the 16- and 32-parent MAGIC populations by DH induction and CS. The construction procedure of the 16- and 32-parent MAGIC populations was similar to that described for the eight-parent MAGIC population. For 16- and 32-parent DH MAGIC populations, the parents were crossed to F_4 and F_5, respectively, and the pure lines were derived by DH induction. For 16- and 32-parent CS MAGIC populations, the parents were crossed to F_4 and F_5, respectively, and continuously self-crossed to obtain the pure lines.

Table S1. Recombination rate variation across all chromosomes under 125 kb, 250 kb and 500 kb windows based on the IBM population.

Chr	125 kb			250 kb			500 kb		
	Min	Max	N	Min	Max	N	Min	Max	N
Chr. 1	0	5.9	2456	0	5.9	1228	0	7.7	614
Chr. 2	0	4.6	1952	0	4.6	976	0	5.1	488
Chr. 3	0	7.2	1888	0	8.1	944	0	8.2	472
Chr. 4	0	4.4	1976	0	7.2	988	0	10.4	494
Chr. 5	0	10.2	1792	0	10.2	896	0	10.2	448
Chr. 6	0	6.3	1392	0	6.4	696	0	7.4	348
Chr. 7	0	12.1	1456	0	12.1	728	0	12.1	364
Chr. 8	0	9.5	1448	0	9.5	724	0	10.1	362
Chr. 9	0	4.2	1280	0	4.9	640	0	6.5	320
Chr. 10	0	5.1	1208	0	5.9	604	0	9.3	302
Total	0	12.1	16848	0	12.1	8424	0	12.1	4212

IBM, intermated B73 and Mo17; N, the total number of recombination bins across all chromosomes; Min and Max represent the minimun and maximum recombination rate of each chromosome, respectively.

Table S2. Estimation of the number of recombination events and recombinant fragment size in DH and RIL populations of different generations.

Generation	Population Type	Number of Recombination Events (per chromosome)	Recombination Fragment Length (Mb)	Total Number of Recombination Bins
2	F_1-DH	14	1.66	1367
3	F_2-DH	21	1.43	1619
4	F_3-DH	25	1.32	1760
5	F_4-DH	26	1.31	1779
6	F_5-DH	26	1.28	1815
7	RIL	27	1.26	1858

Data were obtained under the 125 kb window. Recombination bin denotes block with no recombination in the tested population. Two hundred lines of each population were analyzed.

Table S3. Estimation of the number of recombination events and recombinant fragment size in simulated MAGIC populations derived by the DH induction and continuous self-crossing.

Population Type	Generation	Number of Recombination Events (per chromosome)	Recombination Fragment Length (Mb)	Total number of Recombination Bin
8-parent DH	4	42	1.12	2122
16-parent DH	5	56	1.04	2289
32-parent DH	6	69	0.99	2433
8-parent RIL	8	55	1.03	2314
16-parent RIL	9	69	0.99	2431
32-parent RIL	10	82	0.96	2516

Data were obtained under the 125 kb window. Recombination bin denotes blocks with no recombination in the tested population. Two hundred lines of each population were analyzed.
